# Transcriptomic Analysis of Human Keratinocytes Treated with *Galactomyces* Ferment Filtrate, a Beneficial Cosmetic Ingredient

**DOI:** 10.3390/jcm11164645

**Published:** 2022-08-09

**Authors:** Akiko Nakajima, Nahoko Sakae, Xianghong Yan, Tomohiro Hakozaki, Wenzhu Zhao, Timothy Laughlin, Masutaka Furue

**Affiliations:** 1 Kobe Innovation Center, Procter and Gamble Innovation GK, Kobe 651-0088, Japan; 2 The Procter & Gamble Company, Mason Business Center, Mason, OH 45040, USA; 3 Department of Dermatology, Kyushu University, Fukuoka 812-8582, Japan

**Keywords:** aryl hydrocarbon receptor, claudin 1, claudin 4, chemokine (C-X-C motif) ligand 14, Galactomyces ferment filtrate, interleukin-6 receptor, keratinocyte, small proline-rich proteins 1A and 1B, serpin B2, Pitera™

## Abstract

*Galactomyces* ferment filtrate (GFF, Pitera™) is a cosmetic ingredient known to have multiple skin care benefits, such as reducing redness and pore size via the topical application of its moisturizer form. Although GFF is known to act partly as an antioxidative agonist for the aryl hydrocarbon receptor (AHR), its significance in keratinocyte biology is not fully understood. In this study, we conducted a transcriptomic analysis of GFF-treated human keratinocytes. Three different lots of GFF consistently modulated 99 (22 upregulated and 77 downregulated) genes, including upregulating cytochrome P450 1A1 (*CYP1A1*), a specific downstream gene for AHR activation. GFF also enhanced the expression of epidermal differentiation/barrier-related genes, such as small proline-rich proteins 1A and 1B (*SPRR1A* and *SPRR1B*), as well as wound healing-related genes such as serpin B2 (*SERPINB2*). Genes encoding components of tight junctions claudin-1 (*CLDN1*) and claudin-4 (*CLDN4*) were also target genes upregulated in the GFF-treated keratinocytes. In contrast, the three lots of GFF consistently downregulated the expression of inflammation-related genes such as chemokine (C-X-C motif) ligand 14 (*CXCL14*) and interleukin-6 receptor (*IL6R*). These results highlight the beneficial properties of GFF in maintaining keratinocyte homeostasis.

## 1. Introduction

The maintenance of epidermal homeostasis and structural function is critical for healthy and stress-tolerant skin with a youthful appearance. Facial appearance is an important issue, not only in the elderly, but also in young women [1,2]. Previous studies revealed that skin moisturization is beneficial for keeping a youthful facial appearance [2,3]. A skincare formula containing *Galactomyces* ferment filtrate (GFF, Pitera™) is a functional moisturizing agent, because its topical application was shown to significantly reduce facial erythema, roughness, and pore dilation in two independent clinical trials [2]. In addition, the GFF-containing skincare formula ameliorated the mask-induced exacerbation of facial pore dilation and redness [4].

GFF upregulates the expression of epidermal differentiation complex genes [5] located on chromosome 1q21 [6]. It also ameliorates oxidative stress triggered by various stimuli via the activation of the antioxidative system in keratinocytes [7,8,9,10]. In addition, GFF is known to exert its functional activity, at least in part, as an agonist for the aryl hydrocarbon receptor (AHR) [5,8,9]. However, the detailed activity of GFF on human epidermal keratinocytes remains largely unknown.

In this study, we conducted a transcriptomic analysis of human keratinocytes treated with three different lots of GFF. Compared with the control treatment, all three lots of GFF activated AHR and consistently upregulated the expression of the downstream gene cytochrome P450 1A1 (*CYP1A1*) [5]. The three GFFs also increased the expression of genes encoding AHR-related late-phase epidermal differentiation molecules, such as small proline-rich protein 1A and 1B (*SPRR1A* and *SPRR1B)* [6,11]. In addition to *CYP1A1*, *SPRR1A*, and *SPRR1B*, a significant and meaningful transcriptomic modification was identified in 99 (22 upregulated and 77 downregulated) genes in total. GFFs upregulated the expression of tight junction molecules claudin 1 (*CLDN1*) and claudin 4 (*CLDN4*) [12,13], alternative epidermal differentiation molecules keratin 6 (*KRT6*) and keratin 16 (*KRT16*) [14], and another epidermal differentiation-related molecule, secretory leukocyte peptidase inhibitor (*SLPI*) [15]. In contrast, the three GFFs significantly downregulated the expression of inflammation-related molecules, chemokine C-X-C motif ligand 14 (*CXCL14*) [16,17], and interleukin-6 receptor (*IL6R*) [18,19,20]. These results may explain the molecular basis by which GFF helps to maintain healthy and youthful skin.

## 2. Materials and Methods

### 2.1. Cell Treatment and Sample Preparation for Microarray Analysis

The keratinocyte cell line tKC (tert keratinocytes) was a kind gift from Dr. Jerry W. Shay (University of Texas Southwestern, Dallas, TX, USA) [21,22]. tKC cells were plated at a density of 100,000 cells/well into 12-well plates (Corning BioCoat, REF 354500; Corning Inc., Corning, NY, USA). After growth for 24 h at 37 °C in a CO_2_ incubator, the tKCs were treated with a medium control (10% water) or GFF (10%; P&G Innovation GK, Kobe, Japan) for 24 h before harvesting for microarray analysis. We used three different lots of GFF in this study. Transcriptomic analysis was performed as reported previously [22]. Briefly, samples were collected in RNAlater® buffer, flash-frozen, and stored at −80 °C prior to RNA extraction. RNA was extracted and purified using the RNeasy kit (QIAGEN, Germantown, MD). Purified RNA was converted to biotin-labeled complementary RNA copies using the HT 3′ IVT Plus kit (Affymetrix, Santa Clara, CA, USA), as per the manufacturer’s protocol. Biotinylated cRNA was fragmented using limited alkaline hydrolysis and then hybridized overnight to Affymetrix GeneTitan U219 array plates using the Affymetrix GeneTitan instrument and protocol (ThermoFisher Scientific, Waltham, MA, USA).

### 2.2. Statistical Analysis of Microarray Data

Probe set expression values were calculated with quartile normalization and PLIER summarization algorithms. Differentially expressed genes were analyzed using the empirical Bayes method implemented in the R limma package [23]. *p* values less than 0.001 were considered statistically significant. An increase in gene expression of more than 1.5-fold or a decrease to less than 0.75-fold compared with that of the control treatment was defined as meaningful transcriptomic modification.

## 3. Results

To confirm the functional consistency of GFF, we treated human keratinocytes with three different GFF lots. GFF is known as an AHR agonist [5]. The activation of AHR upregulates the expression of its specific downstream gene *CYP1A1* [5]. AHR activation also upregulates the expression of epidermal differentiation complex genes such as *SPRR1A* and *SPRR1B* [11]. In parallel with the findings in previous studies [5,11], a significant and meaningful upregulation of the *CYP1A1* gene was consistently observed for the three independent lots of GFF (mean fold change: 1.777) (Table 1) compared with the control treatment level. All GFFs also significantly upregulated the expression of *SPRR1A* (mean fold change: 1.647) and *SPRR1B* genes (mean fold change: 2.231) compared with the control treatment levels (Table 1). These results suggested that GFF exerted its AHR agonist activity irrespective of the product lot.

**Figure 1:**
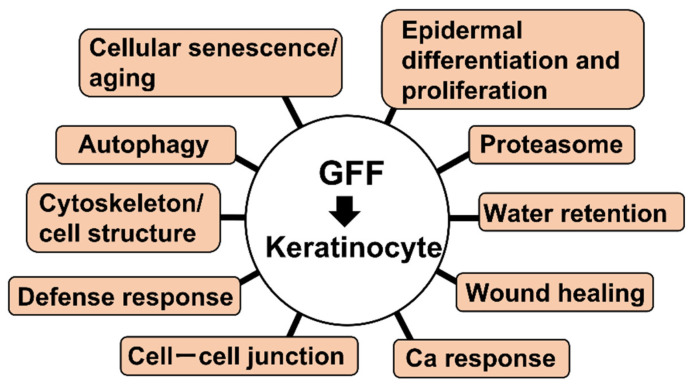
Representative gene groups with modified expression in GFF-treated keratinocytes. GFF: *Galacommyces* ferment filtrate.

In addition to these three upregulated AHR-related genes, the three GFFs consistently up- or downregulated the expression of 96 other genes (19 upregulated and 77 downregulated) (Table 1 and Table 2). Figure 1 and Table 3 list the gene ontogeny (GO) of those genes related to skin.

All three GFF lots significantly increased the expression of genes encoding the tight junction proteins *CLDN1* and *CLDN4* (GO category: establishment of skin barrier), which were previously reported to be upregulated by GFF (Table 1 and Table 3) [5,24]. In the GO category of epidermal differentiation, the expression of KRT6A, KRT6B, and KRT13 was upregulated by the three GFFs (Table 1 and Table 3), while that of the stem cell marker *KRT15* [25] was downregulated (Table 2 and Table 3). As for *KRT6A* and *KRT6B*, the GFFs consistently upregulated the expression of *KRT16*, which is an alternative differentiation marker of epidermal keratinocytes [14] (Table 1 and Table 3).

In the GO category of cell aging, the expression of calreticulin (*CALR*) was downregulated, whereas that of *PLK2* was upregulated (Table 1 and Table 3). Meanwhile, the expression of the genes *COL7A1*, *DLL1*, and *WNT10A* (GO category: epidermis development); *DDX60*, *DHX58*, and *PSMB9* (positive regulation of defense response); *DNAJB9*, *HERPUD1*, *HSP90B1*, *SDF2L1*, and *SEL1L* (proteasomal protein catabolic process); *GJB2*, *HEG1*, and *MICALL2* (cell–cell junction assembly); *SYT8* (cellular response to calcium ion); and *DST* (microtubule-based movement) was consistently decreased by GFF (Table 2 and Table 3). In contrast, the expression of the genes *GAL* (epidermis development) and *SERPINB2* (regulation of wound healing) was augmented by GFF. In addition to *SERPINB2*, GFF also upregulated the expression of the *SERPINB1* and *SERPINB7* genes (Table 1 and Table 3). However, the biological significance of the modified expression of these genes in keratinocytes remains obscure.

**Figure 2:**
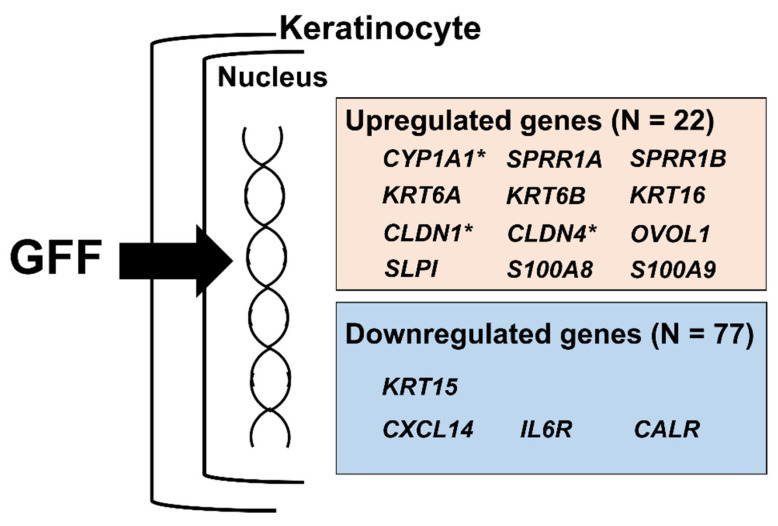
Representative genes with modified expression in GFF-treated keratinocytes. * Genes which were reported to be modified in GFF-treated keratinocytes. GFF: *Galactomyces* ferment filtrate.

The gene expression of the secretory leukocyte peptidase inhibitor (*SLPI*) [15] is also known to be related to epidermal differentiation. Similar to the abovementioned epidermal differentiation genes, GFF significantly upregulated the expression of the *SLPI* gene (Table 1). In contrast, the expression of the epidermal proliferation-related gene fibroblast growth factor receptor 3 (*FGFR3*) [26,27] was downregulated by GFF (Table 3). In addition, GFF was likely to ameliorate the inflammatory process, because it strongly inhibited the expression of the *CXCL14* [16,17], *IL6R* [18,19], and *CALR* [20] genes (Table 3). Representative genes for which the expression was modified in the GFF-treated keratinocytes are depicted in Figure 2.

Finally, when we set a less stringent threshold for defining significantly modulated genes to an increase in expression of more than 1.2-fold or a decrease to less than 0.8-fold compared with the control, 175 upregulated and 20 downregulated genes were added as target genes modulated by GFF, including S100A8, S100A9, and OVOL1 (Supplementary Tables S1 and S2). Notably, the expression of these three genes is known to be upregulated by AHR activation [28,29,30].

## 4. Discussion

The GFF-formulated moisturizing product is a popular skincare product used widely around the world. Two independent clinical trials have shown that its daily application for 4 weeks significantly attenuated not only the intensity, but also the fluctuation of facial redness, roughness, and pore dilation [2]. Topical GFF also stabilized the mask-induced exacerbation of fluctuations in facial redness and pore dilation [4]. The clinical efficacy of GFF may be partly attributable to the fact that it works as an antioxidative AHR agonist [5,7,8,9,10]. However, the molecular effects of GFF on keratinocytes are not fully understood.

In the present study, we performed the transcriptomic analysis of human keratinocytes treated with three different lots of GFF. In accordance with previous studies [5,11], all three GFFs significantly upregulated the expression of *CYP1A1*, *SPRR1A*, and *SPRR1B*, which are known downstream genes of AHR activation. These results confirmed the AHR agonist activity of GFF, irrespective of the product lot. CYP1A1 may be useful for degrading environmental pollutants [31], while SPRR1A and SPRR1B are important epidermal barrier molecules [6]. In parallel, GFF upregulated the expression of other AHR-mediated genes, such as *S100A8*, *S100A9*, and *OVOL1*. S100A8 and S100A9 form a heterodimer called calprotectin, which works as a keratinocyte alarmin molecule [32]. OVOL1 is a transcription factor essentially involved in the induction of barrier-related proteins [29,30]. In addition, all three GFF lots in this study consistently upregulated the expression of *CLDN1* and *CLDN4*, as reported previously [5,24]. These results suggested that GFF may enhance or accelerate barrier formation (SPRR1A and SPRR1B) and tight junction formation (CLDN1 and CLDN4).

The accelerating activity of GFF on epidermal differentiation or barrier formation can be further highlighted by the fact that it also upregulated the expression of *SLPI*. SLPI expression is reported to be upregulated in the cornified layer by antioxidative signaling and is related to the desquamation process [15]. Notably, SLPI is also known as an endogenous ligand for the annexin A2 heterotetramer, which serves as an uptake receptor for human papilloma virus in keratinocytes [33]. The blocking of the annexin A2 heterotetramer by SLPI inhibits the human papilloma virus infection [33]. In contrast to the differentiation-prone gene response, GFF is likely to inhibit the proliferation of keratinocytes via the downregulation of *FGFR3* expression. FGFR3 plays a crucial role in keratinocyte proliferation, because the gain-of-function mutation of *FGFR3* causes the development of epidermal nevi [26,27].

Various chemical and mechanical injuries induce the expression of the alternative differentiation keratin pair KRT6/KRT16 [14,34,35]. Recent studies have revealed that KRT6 and KRT16 act as key early barrier alarmins and upregulate the stress response and innate immunity [34,35]. The present study clearly demonstrated that GFF was a potent inducer of *KRT6*/*KRT16* barrier alarmins. In contrast, KRT15 is recognized as a useful marker of epidermal keratinocytic stem cells [25]. Notably, in the present study, GFF significantly and potently downregulated *KRT15* expression. We speculated that GFF may accelerate epidermal keratinocyte differentiation partly through enhancing the exit from stemness by downregulating *KRT15*.

The GFF-mediated downregulation of *CXCL14*, *IL6R*, and *CALR* may underscore the immunoregulatory function of GFF. CXCL14 is a potent chemoattractant of immune cells, especially monocytes and dendritic cells [16,17]. The proinflammatory cytokine IL-6 is produced in keratinocytes facing barrier disruption or chemicals [18,19], and is related to eczematous dermatitis [20]. Meanwhile, CALR has recently been recognized as an inducer of immunogenic cell death [36] and is critically involved in programmed cell removal by macrophages [37,38].

GFF (Pitera™) is a quality-assured, filtrated material derived from *Galactomyces* fermentation. It consists of over 50 components, including minerals, vitamins, amino acids, and organic acids. As shown in the present study, three different lots of GFF consistently revealed similar transcriptomic effects on human keratinocytes. There were several limitations to this study. First, no proteomic analysis was performed here, so this needs to be carried out in future work to confirm the present transcriptomic results. As GFF is a mixture of active substances derived from *Galactomyces*, it is not surprising that it acts on many different targets additively or synergistically. Second, although a meaningful transcriptomic alteration was identified in 99 genes, the roles of most of these genes in keratinocyte biology are not fully understood. Therefore, further studies are warranted to reveal the implications of these genes in keratinocyte homeostasis. Third, the dependence of the 99 genes on AHR remains largely unknown. For example, as mentioned above, AHR regulates the gene expression of *CYP1A1*, *SPRR1A*, and *SPRR1B* [5,11], but that of *CLDN1* and *CLDN4* is not dependent on AHR activation [5].

In conclusion, this study showed that GFF is a biologically active cosmetic ingredient that serves as an AHR agonist irrespective of the particular product lot. GFF appeared to increase the expression of differentiation/barrier-related genes (*SPRR1A*, *SPRR1B*, *CLDN1*, *CLDN4*, and *SLPI*), but decreased that of a proliferation-related gene (*FGFR3*) in keratinocytes. It also upregulated the barrier alarmin genes (*KRT6* and *KRT16*), while downregulating a stemness gene (*KRT15*). In addition, GFF likely ameliorated the inflammatory process by downregulating the expression of the *CXCL14*, *IL6R*, and *CALR* genes. The coordinated regulation of these genes may underpin the beneficial activity of GFF in maintaining healthy skin.

## Figures and Tables

**Table 1:** Genes upregulated ^#^ by different lots of GFF.

Gene	GFF Lot			Gene	GFF Lot		
	Lot 1	Lot 2	Lot 3		Lot 1	Lot 2	Lot 3
*KRT13*	2.357 *	2.488	2.351	*KRT6A*	1.684	1.842	1.693
*PI3*	2.310	2.439	2.224	*CLDN1*	1.717	1.659	1.619
*SPRR1B*	2.247	2.382	2.063	*GAL*	1.600	1.702	1.669
*KRT6B*	2.123	2.152	2.120	*SERPINB7*	1.645	1.656	1.667
*UPK1B*	1.957	2.082	1.955	*KRTAP2*	1.664	1.555	1.736
*SERPINB2*	1.920	1.962	1.962	*SPRR1A*	1.525	1.814	1.601
*PLK2*	1.981	1.915	1.847	*GPRC5A*	1.763	1.590	1.582
*CTSC*	1.920	1.936	1.729	*MAL2*	1.646	1.709	1.552
*KRT16*	1.736	1.904	1.709	*ZNF750*	1.542	1.740	1.602
*CYP1A1*	1.525	2.208	1.597	*CLDN4*	1.639	1.627	1.517
*SERPINB1*	1.791	1.753	1.697	*SLPI*	1.545	1.572	1.520

^#^ All genes were significantly upregulated by GFF (*p* value < 0.001). * Fold change. More than 1.5-fold upregulation compared with the control is considered meaningful. GFF: *Galactomyces* ferment filtrate.

**Table 2:** Genes downregulated ^#^ by different lots of GFF.

Gene	GFF Lot			Gene	GFF Lot		
	Lot 1	Lot 2	Lot 3		Lot 1	Lot 2	Lot 3
*CXCL14*	0.244 *	0.257	0.302	*TSC22D3*	0.628	0.703	0.667
*HERPUD1*	0.444	0.416	0.447	*ISG15*	0.644	0.664	0.696
*LTB*	0.460	0.402	0.460	*MIR4680*	0.659	0.675	0.671
*HSPA5*	0.481	0.456	0.504	*FILIP1L*	0.669	0.658	0.685
*KRT15*	0.507	0.471	0.545	*PRKCDBP*	0.685	0.642	0.694
*CALR*	0.556	0.497	0.528	*RAB7B*	0.656	0.686	0.682
*GLUL*	0.501	0.544	0.559	*PDIA3*	0.669	0.684	0.684
*LGALS7*	0.494	0.559	0.562	*WNT10A*	0.657	0.679	0.703
*MANF*	0.548	0.552	0.561	*ZBTB16*	0.645	0.674	0.724
*VAV3*	0.539	0.555	0.570	*DST*	0.698	0.653	0.696
*CRELD2*	0.559	0.546	0.559	*IMPA2*	0.653	0.698	0.699
*PDIA4*	0.549	0.555	0.563	*TNS3*	0.663	0.667	0.720
*IFIT1*	0.556	0.579	0.577	*P4HB*	0.667	0.686	0.698
*AHNAK2*	0.568	0.562	0.612	*DDX60*	0.696	0.670	0.686
*IFITM1*	0.565	0.581	0.600	*ST6GALNAC2*	0.684	0.698	0.681
*HSP90B1*	0.593	0.571	0.590	*HEG1*	0.691	0.683	0.708
*NUCB2*	0.581	0.564	0.610	*MICALL2*	0.685	0.716	0.682
*PNRC1*	0.578	0.574	0.612	*IFIT2*	0.708	0.699	0.685
*SDF2L1*	0.593	0.578	0.595	*IFI44*	0.682	0.714	0.698
*SULF2*	0.553	0.589	0.631	*ITGB8*	0.675	0.705	0.723
*FLRT2*	0.599	0.578	0.605	*SOX6*	0.683	0.690	0.741
*FGFR3*	0.563	0.628	0.636	*PIK3R1*	0.683	0.702	0.742
*COL7A1*	0.596	0.615	0.633	*CLCA2*	0.710	0.703	0.731
*METTL7A*	0.582	0.625	0.644	*SMIM14*	0.692	0.746	0.714
*PRSS23*	0.602	0.594	0.665	*C1R*	0.718	0.723	0.714
*ASS1*	0.620	0.605	0.638	*DNAJB9*	0.737	0.706	0.713
*HYOU1*	0.612	0.616	0.646	*LGALS1*	0.700	0.716	0.741
*DLL1*	0.648	0.622	0.648	*SEL1L*	0.733	0.705	0.722
*GJB2*	0.616	0.625	0.683	*PBX1*	0.701	0.727	0.742
*TNNI2*	0.556	0.660	0.717	*PSMB9*	0.693	0.744	0.745
*PDIA6*	0.642	0.645	0.651	*PPIB*	0.729	0.713	0.742
*HTRA1*	0.622	0.663	0.655	*IL6R*	0.725	0.725	0.741
*SYT8*	0.605	0.665	0.684	*ETS2*	0.719	0.742	0.731
*DLK2*	0.655	0.629	0.672	*LFNG*	0.726	0.724	0.745
*CDK2AP2*	0.651	0.656	0.652	*DHX58*	0.723	0.734	0.744
*ACKR3*	0.635	0.622	0.705	*OLFML2A*	0.743	0.723	0.736
*TGFBI*	0.578	0.700	0.689	*TMEM50B*	0.749	0.722	0.738
*IRF9*	0.652	0.652	0.673	*AGR2*	0.748	0.744	0.734
*TNFRSF21*	0.613	0.693	0.690				

^#^ All genes were significantly downregulated by GFF (*p* value < 0.001). * Fold change. Downregulation to less than 0.75-fold compared with the control is considered meaningful. GFF: *Galactomyces* ferment filtrate.

**Table 3:** Gene ontology of skin-related genes up- and downregulated by GFF.

Group	Term	Gene	tKC RNA Expression
**Cellular senescence/aging**	GO:0090342 regulation of cell aging	*PLK2*	Up
	GO:0090398 cellular senescence GO:0007569 cell aging	*PLK2*	Up
		*CALR*	Down
**Autophagy**	GO:0006914 autophagy GO:0010508 positive regulation of autophagy	*PLK2*	Up
**Cytoskeleton/cell structure**	GO:0000226 microtubule cytoskeleton organization	*PLK2*	Up
		*CDK2AP2, DST*	Down
	GO:0007015 actin filament organization	*HSP90B1, MICALL2, PIK3R1*	Down
	GO:0007018 microtubule-based movement GO:0045104 intermediate filament cytoskeleton organization	*DST*	Down
**Defense response**	GO:0031349 positive regulation of defense response	*CTSC*	Up
		*DDX60, DHX58, PSMB9*	Down
**Cell–cell junction**	GO:0007043 cell–cell junction assembly	*CLDN1*	Up
		*GJB2, HEG1, MICALL2*	Down
	GO:0007044 cell–substrate junction assembly	*DST*	Down
	GO:0120192 tight junction assembly	*CLDN1*	Up
		*MICALL2*	Down
**Proteasome**	GO:0010498 proteasomal protein catabolic process	*PLK2*	Up
		*DNAJB9, HERPUD1, HSP90B1, HSPA5, PSMB9, SDF2L1, SEL1L*	Down
	GO:0043161 proteasome-mediated ubiquitin-dependent protein catabolic process	*PLK2*	Up
		*DNAJB9, HERPUD1, HSP90B1, HSPA5, PSMB9, SEL1L*	Down
**Epidermal differentiation and proliferation**	GO:0008544 epidermis development	*GAL, KRT6A, KRT6B, KRT13, PI3, SPRR1A, SPRR1B, ZNF750*	Up
		*COL7A1, DLL1, KRT15, WNT10A*	Down
	GO:0009913 epidermal cell differentiation	*KRT6A, KRT6B, KRT13, PI3, SPRR1A, SPRR1B*	Up
		*DLL1, KRT15*	Down
	GO:0030216 keratinocyte differentiation GO:0031424 keratinization	*KRT6A, KRT6B, KRT13, PI3, SPRR1A, SPRR1B*	Up
		*KRT15*	Down
	GO:0033561 regulation of water loss via skin GO:0061436 establishment of skin barrier	*CLDN1, CLDN4*	Up
	GO:0070268 cornification	*KRT6A, KRT6B, KRT13, PI3, SPRR1A, SPRR1B*	Up
		*KRT15*	Down
**Water retention**	GO:0030104 water homeostasis	*CLDN1, CLDN4*	Up
**Wound healing**	GO:0061041 regulation of wound healing	*SERPINB2*	Up
**Ca response**	GO:0071277 cellular response to calcium ion	*HSPA5, SYT8*	Down

Up: More than 1.5-fold upregulation compared with the control is considered meaningful. Down: Downregulation to less than 0.75-fold compared with the control is considered meaningful. GFF: *Galactomyces* ferment filtrate.

## Data Availability

The data presented in this study are available on request from the corresponding author. The data are not publicly available because of institutional restrictions.

## Supplementary Materials

The following supporting information can be downloaded at: https://www.mdpi.com/article/10.3390/jcm11164645/s1, Table S1: Genes upregulated# by different lots of GFF (1.2–1.5-fold); Table S2: Genes downregulated# by different lots of GFF (0.75–0.8-fold).
