# Supplementary material for: Transcriptomic Analysis of Human Keratinocytes Treated with Galactomyces Ferment Filtrate, a Beneficial Cosmetic Ingredient
Source: J Clin Med. 2022 Aug 9;11(16):4645. doi: 10.3390/jcm11164645 (PMC9409768; doi:10.3390/jcm11164645)
Supplement: Supplementary file 1 [file jcm-11-04645-s001.zip › jcm-1828756-supplementary.pdf]

Supplementary Table S1. Genes upregulated[#] by different lots of GFF (1.2–1.5-fold)

Gene	Lot 1	Lot 2	Lot 3	Gene	Lot 1	Lot 2	Lot 3	Gene	Lot 1	Lot 2	Lot 3
LYAR	1.452	1.474	1.443	SLC25A15	1.304	1.314	1.323	ESF1	1.289	1.215	1.267
GRHL3	1.421	1.472	1.423	TPD52L1	1.350	1.334	1.254	ATAD3A	1.288	1.223	1.254
PLAUR	1.476	1.424	1.382	CRCT1	1.359	1.322	1.252	MRTO4	1.226	1.258	1.281
HS3ST1	1.467	1.441	1.368	CTPS1	1.297	1.362	1.271	CMTM7	1.260	1.254	1.249
S100A8	1.454	1.390	1.427	KRT8	1.350	1.276	1.302	RABGGTB	1.275	1.223	1.260
ID3	1.378	1.432	1.445	PNPT1	1.297	1.328	1.301	IFRD2	1.209	1.262	1.282
S100A9	1.413	1.383	1.431	EMP1	1.339	1.303	1.282	NOP2	1.202	1.254	1.298
METRNL	1.351	1.447	1.408	NDUFAF4	1.311	1.318	1.293	RRP9	1.269	1.231	1.247
NOLC1	1.469	1.402	1.333	POLR1B	1.310	1.310	1.288	COA4	1.257	1.243	1.247
DDX21	1.398	1.380	1.418	C1orf74	1.322	1.272	1.314	MIR1292	1.233	1.249	1.265
PNO1	1.387	1.405	1.394	RPF2	1.333	1.280	1.289	CRABP2	1.259	1.262	1.223
NOP16	1.446	1.364	1.370	PPAN	1.355	1.301	1.244	ISG20L2	1.229	1.251	1.264
MYC	1.357	1.422	1.374	TAGLN	1.329	1.294	1.275	SNORD17	1.267	1.229	1.247
LAMA2	1.443	1.376	1.333	ADRB2	1.321	1.298	1.276	PDCD11	1.220	1.262	1.257
MACC1	1.369	1.426	1.352	SLC25A32	1.351	1.283	1.261	PA2G4	1.230	1.244	1.261
OVOL1	1.433	1.394	1.317	A2ML1	1.316	1.325	1.250	PFDN2	1.223	1.256	1.253
CCND1	1.358	1.394	1.391	BYSL	1.241	1.320	1.330	RFK	1.265	1.233	1.232
RRS1	1.421	1.396	1.320	ZMYND19	1.325	1.255	1.307	MYBBP1A	1.283	1.237	1.206
IL1R2	1.441	1.350	1.321	PHLDA2	1.334	1.283	1.267	AKAP2	1.263	1.207	1.251
IL1B	1.373	1.403	1.332	LTV1	1.298	1.293	1.290	C19orf48	1.269	1.238	1.213
KRT34	1.407	1.402	1.298	NRG1	1.295	1.300	1.278	BCCIP	1.264	1.234	1.222
THBS1	1.372	1.351	1.382	DKC1	1.355	1.274	1.244	METAP2	1.248	1.219	1.252
GADD45A	1.355	1.376	1.374	LOX	1.358	1.253	1.261	BCL2L1	1.261	1.211	1.247
KCTD12	1.377	1.330	1.395	CIRH1A	1.261	1.310	1.300	SLC25A33	1.227	1.267	1.225
IL36G	1.357	1.396	1.348	HES2	1.226	1.349	1.295	SCD5	1.213	1.241	1.262
SNX5	1.432	1.361	1.307	CCDC86	1.294	1.287	1.289	TRMT6	1.262	1.230	1.216
WDR3	1.374	1.360	1.366	GNL3	1.269	1.314	1.284	MRPS30	1.241	1.241	1.223
PVRL4	1.344	1.412	1.331	DBNDD2	1.284	1.314	1.269	PTRH2	1.254	1.206	1.242
POLR3G	1.412	1.343	1.327	RPS14	1.352	1.279	1.233	SLC2A14	1.241	1.213	1.248
GCLM	1.365	1.384	1.320	RND3	1.344	1.243	1.273	TM4SF1	1.218	1.241	1.243
HES1	1.331	1.382	1.350	EXOSC4	1.274	1.316	1.270	NAT10	1.241	1.232	1.221
BRIX1	1.352	1.352	1.359	KIAA0020	1.283	1.296	1.275	NUDCD1	1.254	1.210	1.229
TGM1	1.412	1.394	1.257	FAM136A	1.252	1.289	1.313	NMD3	1.254	1.236	1.202
DUSP5	1.376	1.320	1.362	CCNB1IP1	1.268	1.338	1.245	MINA	1.249	1.231	1.211
PROSER2	1.367	1.355	1.334	TSR1	1.298	1.275	1.270	NTMT1	1.213	1.274	1.202
DUSP4	1.390	1.344	1.320	GRPEL1	1.327	1.273	1.243	GART	1.270	1.202	1.217
FABP5	1.289	1.352	1.410	RFC3	1.309	1.275	1.255	TIPIN	1.218	1.233	1.239
TXNRD1	1.397	1.385	1.267	SLC20A1	1.344	1.270	1.223	MRPS17	1.227	1.254	1.205
SBSN	1.300	1.413	1.321	SFXN4	1.268	1.296	1.268	BID	1.254	1.208	1.222
SCEL	1.359	1.342	1.332	PAK1IP1	1.317	1.275	1.239	PPRC1	1.235	1.204	1.245
FAM25A	1.351	1.401	1.279	EIF3B	1.297	1.257	1.268	WDR74	1.208	1.268	1.206
SRM	1.339	1.311	1.359	PROCR	1.242	1.319	1.247	IL1RN	1.207	1.220	1.254
ADGRF1	1.375	1.363	1.263	AMOTL2	1.234	1.259	1.311	SNHG1	1.219	1.224	1.235
FKBP4	1.303	1.351	1.339	ARRDC4	1.211	1.323	1.269	CSNK1E	1.247	1.223	1.206
CXorf49	1.295	1.332	1.360	NSUN2	1.315	1.255	1.233	TACSTD2	1.236	1.225	1.205
B4GALT4	1.327	1.345	1.314	PUS1	1.311	1.269	1.218	UMPS	1.220	1.235	1.206
ERVMER34-1	1.346	1.311	1.318	MRPS2	1.284	1.266	1.245	HSPD1	1.235	1.207	1.218
PDCD2L	1.297	1.370	1.308	TRAP1	1.278	1.276	1.237	ALAS1	1.239	1.212	1.206
UPP1	1.382	1.289	1.299	RIOK1	1.264	1.241	1.287	EPHA2	1.222	1.207	1.226
SPRR2D	1.330	1.370	1.268	TFRC	1.356	1.221	1.213	PITPNC1	1.202	1.226	1.227
SLC25A19	1.305	1.336	1.326	RPL27A	1.256	1.255	1.276	TEX10	1.227	1.220	1.207
GTPBP4	1.344	1.329	1.291	OCLN	1.259	1.268	1.261	PRPF4	1.223	1.216	1.214
YRDC	1.348	1.308	1.304	NIP7	1.268	1.266	1.252	ASUN	1.210	1.219	1.220
JPH1	1.322	1.342	1.296	RSL1D1	1.250	1.235	1.295	POLR1E	1.213	1.231	1.205
GEMIN5	1.272	1.326	1.356	WDR77	1.265	1.244	1.269	RUVBL1	1.203	1.202	1.239
SERPINB13	1.242	1.395	1.316	VGLL3	1.312	1.223	1.242	EIF3J	1.220	1.211	1.203
WDR43	1.350	1.300	1.302	TXNIP	1.213	1.253	1.310	GNL2	1.203	1.213	1.206
GPATCH4	1.367	1.319	1.265	SDCCAG3	1.240	1.286	1.247	NLN	1.207	1.203	1.203
MARS2	1.376	1.288	1.277								

#All genes are significantly upregulated by GFFs (P value < 0.001). Genes upregulated more than 1.2- to 1.5-fold compared with the control are listed.

Supplementary Table S2. Genes downregulated# by different lots of GFF (0.75–0.8-fold)

	Lot 1	Lot 2	Lot 3
ITGAV	0.754	0.754	0.766
CA12	0.777	0.752	0.762
DNER	0.776	0.763	0.755
P4HA2	0.760	0.770	0.766
ITGB6	0.751	0.788	0.758
ERAP1	0.778	0.763	0.768
ECH1	0.762	0.759	0.798
PDGFC	0.762	0.770	0.791
KANK2	0.780	0.756	0.794
ERAP2	0.763	0.782	0.793
ZNF395	0.772	0.768	0.799
SSBP3	0.765	0.797	0.778
TP63	0.780	0.766	0.794
FBXO16	0.760	0.784	0.796
DNAJB11	0.782	0.774	0.785
SECTM1	0.753	0.797	0.792
CREB3L2	0.782	0.774	0.791
CROCC	0.800	0.781	0.781
TLL1	0.796	0.787	0.789
SLITRK6	0.799	0.796	0.792

#All genes are significantly downregulated by GFFs (P value < 0.001).
Genes downregulated to less than 0.8- to 0.75-fold compared with the control are listed.
